# Basidiobolomycosis Simulating a* Mycobacterium ulcerans* Infection in a Togolese Rural Child

**DOI:** 10.1155/2017/6905783

**Published:** 2017-10-17

**Authors:** Bayaki Saka, Waguena Gnassingbe, Garba Mahamadou, Sefako Akakpo, Julienne Teclessou, Aurel Abilogun-Chokki, Abas Mouhari-Toure, Koussake Kombate, Palokinam Pitché

**Affiliations:** ^1^Service de Dermatologie et IST, CHU Sylvanus Olympio, Université de Lomé, Lomé, Togo; ^2^Service de Dermatologie et IST, CHU Campus de Lomé, Université de Lomé, Lomé, Togo; ^3^Service de Dermatologie et IST, CHU de Kara, Université de Kara, Kara, Togo

## Abstract

**Background:**

Basidiobolomycosis is a deep mycosis which preferentially affects rural young people in tropical countries. We report an atypical case, with multiple ulcers, simulating a Buruli ulcer.

**Case Report:**

A 5-year-old boy, living in a rural area, was seen for ulcers on the buttocks and at the back and right flank that had been in progress for 4 months. On examination, we found an infiltrated plaque with sharp edges, little painful, located on the buttocks, back, and the right flank. On this plaque, there were multiple ulcers with polycyclic contours and fibrinous bottom. There were inguinal inflammatory lymph nodes. The patient had an altered general condition. Examination of other organs was normal. The diagnosis of Buruli ulcer was evoked first; the search for* Mycobacterium ulcerans* by polymerase chain reaction was negative. Histology test performed revealed hypodermic granulomatous inflammation with predominant macrophage and eosinophils. The mycological culture was not done. The child was treated successfully with ketoconazole (10 mg/kg/day) during eight weeks.

**Discussion:**

Our observation shows great clinical and epidemiological similarities between basidiobolomycosis and Buruli ulcer. It confirms the efficacy of ketoconazole in severe basidiobolomycosis infection with alteration of general condition. Histopathology is very important for differential diagnosis between these two diseases.

## 1. Introduction

Basidiobolomycosis is a rare childhood mycosis which preferentially affects rural young people in tropical countries mainly in Africa, Asia, and Latin America [1–5]. The disease is most often clinically recognized by the presence of one or more well-defined, homogeneous, dermohypodermal plaques, movable in relation to the deep plane and essentially located at the root of the limbs [1, 2, 6, 7]. We report an atypical case of basidiobolomycosis with multiple ulcers, simulating a Buruli ulcer in a Togolese rural child.

## 2. Case Report

**Figure 1:**
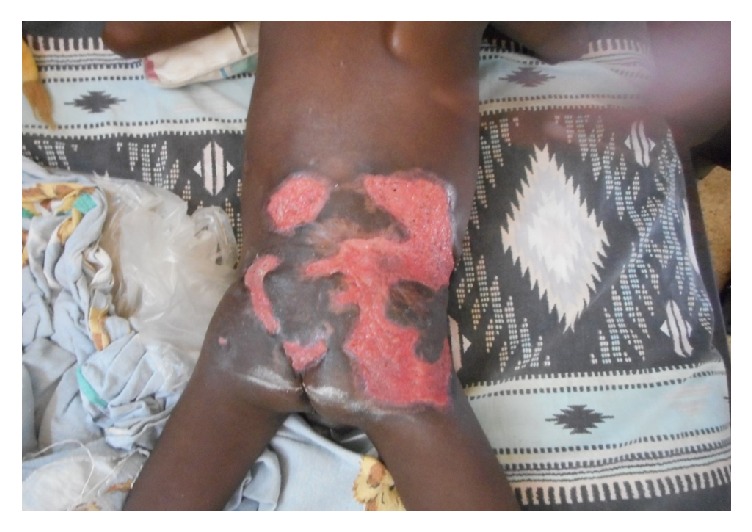
Basidiobolomycosis with ulcers in the buttocks.

A 5-year-old boy, living in a rural area, was seen in a dermatology unit for ulcers in the buttocks, at the back and right flank that had been evolving for 4 months. The lesions began 17 months earlier with a painless nodule in the right lumbar fossa which evolved in two months to a painless plaque extending to the back and right flank and then ulcerated. The child was completely vaccinated. There was no history of preexisting wound on the back and right flank of the child. On physical examination, we found an infiltrated plaque, with sharp edges, little painful, located on the buttocks, back, and the right flank. On this plaque, there were multiple hollow ulcers with polycyclic contours and fibrinous bottom (Figure 1). There were inguinal inflammatory lymph nodes. The patient had an altered general condition and was confined to bed. Physical examination of other organs was normal. The diagnosis of Buruli ulcer was discussed, but polymerase chain reaction test for* Mycobacterium ulcerans* was negative. Histology test performed revealed hypodermic granulomatous inflammation with predominant macrophage and eosinophils. The mycological culture to identify the germ was not done because of technical reasons. The patient was treated with ketoconazole (10 mg/kg/day). Transaminases levels were assessed at the beginning of treatment and every two weeks during the treatment. The development was favorable after eight weeks of treatment with regression of the plaque followed by healing of the ulcers.

## 3. Discussion

We report a second case of basidiobolomycosis simulating a* Mycobacterium ulcerans* infection in a Togolese young boy living in a rural area. This second observation confirms the efficacy of ketoconazole (10 mg/kg/day) for eight weeks even in case of severe basidiobolomycosis infection with alteration of general condition. Indeed, the main difference between the current basidiobolomycosis case and the earlier reported case [7] is the general condition status of the current case which was altered and the patient being confined to bed. Moreover, there was a difference in the location of the wounds: buttocks, back, and flank in this current observation, while in the earlier reported case wounds were located on the chest and neck. Although culture was not performed, the case was classified as a basidiobolomycosis based on epidemiological, clinical, and histopathological arguments and the efficacy of antifungal drug administered. Also, there is no reported history of preexisting wound that got worse with pyogenic infectious agent. This observation raises the difficulty of differential diagnosis between Buruli ulcer and basidiobolomycosis in rural setting in tropical zone. This difficulty explains the confusion that had occurred in a previous case where a child's limb was amputated by misdiagnosis [7]. Other authors have reported cases of basidiobolomycosis with ulcer simulating a Buruli ulcer elsewhere [8–10]. Indeed, there is a resemblance between these two affections on epidemiological aspects (diseases encountered in rural areas especially in children in tropical areas) as well as on clinical aspects (cardboard dermohypodermic plaques, homogeneous well limited, mobile with respect to the deep plan for the basidiobolomycosis that can secondarily be ulcerated versus nodular or oedematous onset before the ulcer for Buruli ulcer). But the first distinction is related to the location of the lesions: especially the root of the limbs for basidiobolomycosis versus the areas in contact with the soil (especially the limbs, buttocks) for Buruli ulcer. Alteration of general condition is not common during* Mycobacterium ulcerans* infection, except complications. The second difference stems from the fact that basidiobolomycosis is a deep mycosis, forming with conidiobolomycosis the family of Entomophthoromycosis. The etiologic agent is* Basidiobolus ranarum* [11], a saprophyte in the soil and plant of tropical and subtropical countries. This fungus belongs to the class of phycomycetes, to the family of Entomophthoraceae, to the order of Entomophthorales and to the genus* Basidiobolus*. In contrast, Buruli ulcer is a necrotizing infection of subcutaneous fatty tissue caused by* Mycobacterium ulcerans* that occurs in more than 30 countries in Africa, Latin America, Oceania, and Asia [12]. Histopathology is very important in the diagnosis of this affection, especially in tropical countries where it may simulate* Mycobacterium ulcerans* infection. It reveals in the case of basidiobolomycosis an hypodermic granulomatous inflammation with predominant macrophage and eosinophils with a Splendore Hoppli phenomenon. The histology of Buruli ulcer shows extensive necrosis without casein and a disappearance of adipocytes nuclei and fibrinous sediments around fat lobules, with, at the center of the necrosis, the acid-resistant bacilli after Ziehl-Nielsen coloration.

This case confirms the efficacy of azole derivatives in the treatment of basidiobolomycosis. We treated our patient with ketoconazole at a dose of 10 mg/kg/day, with good progress after 6 months. This drug is very effective and well tolerated [7, 13], in contrast to potassium iodide and cotrimoxazole which are less effective and less tolerated. Other azole derivatives such as itraconazole, fluconazole, and terbinafine have been used successfully during basidiobolomycosis.

## 4. Conclusion

This observation shows the necessity to think of basidiobolomycosis in case of wound in a child living in a rural area, whatever the location of the wounds, with or without alteration of the general condition of the patient. Ketoconazole, for the same posology, remains a very effective and well tolerated treatment, even in case of severe basidiobolomycosis infection with alteration of general condition.

## Additional Points

*Limitations*. The mycological culture to identify the germ was not done because of technical reasons.
